# STF-DKANMixer: Tri-component decomposition with KAN-MLP hybrid architecture for time series forecasting

**DOI:** 10.1371/journal.pone.0337793

**Published:** 2025-12-08

**Authors:** Junxiang Wei, Rongzuo Guo, Yuning Wang

**Affiliations:** 1 College of Computer Science, Sichuan Normal University, Chengdu, China; 2 Academic Affairs Office, Sichuan Water Conservancy Vocational College, Chongzhou, China; Buckinghamshire New University - High Wycombe Campus: Buckinghamshire New University, UNITED KINGDOM OF GREAT BRITAIN AND NORTHERN IRELAND

## Abstract

Long-term time series forecasting is critical for domains such as traffic and energy systems, yet contemporary models often fail to capture complex multiscale patterns and nonlinear dynamics, resulting in significant inaccuracies during periods of abrupt change. To overcome these limitations, we introduce **STF-DKANMixer**, a novel hybrid architecture combining a Multi-Layer Perceptron (MLP) with the expressive power of the Kolmogorov–Arnold Network (KAN). Our framework begins with a DFT-based decomposition strategy: long-term trends and seasonal components are extracted directly via Discrete Fourier Transform (DFT), while the remaining residual is further decomposed into high-frequency details using a Haar wavelet transform with error compensation. In the **Past-Information-Mixing (PIM)** stage, each component is processed by a GELU-activated KAN module for superior nonlinear feature mapping before being fused by a novel deformable feature attention (DFA) block, which adaptively learns sampling offsets and weights to capture complex dependencies. Subsequently, the **Future-Information-Mixing (FIM)** stage leverages an adaptive weighted ensemble of multiple lightweight predictors, enhanced by residual connections, to generate the final forecast. Extensive experiments on benchmark datasets validate the superiority of our approach. STF-DKANMixer significantly outperforms state-of-the-art models, reducing Mean Squared Error (MSE) by up to **36.1%** (12.3% on average) and Mean Absolute Error (MAE) by up to **28.8%** (8.8% on average). Impressively, these results are achieved while using less than half the computational resources of comparable methods. Our findings establish STF-DKANMixer as a robust, efficient, and highly accurate solution, setting a new performance standard for complex, long-horizon forecasting tasks.

## 1 Introduction

Time series forecasting plays a pivotal role in data science and engineering, with widespread applications in fields such as economics [1,2], energy [3,4], traffic flow planning [5,6], and weather forecasting [7]. The fundamental objective of time series forecasting is to infer future trends based on historical observations [8]. However, real-world time series are often highly complex and non-stationary, exhibiting a variety of intertwined dynamic patterns—such as upward and downward trends, abrupt fluctuations, and irregular cycles—that pose significant challenges for accurate prediction. These challenges are further exacerbated by the presence of noise, missing values, and external factors that can influence the observed data. Accurately capturing the underlying regularities in such complex and variable time series data is not only of great theoretical value but also has profound practical implications, as improved forecasting can lead to better decision-making in critical domains. Thus, conducting in-depth research on forecasting complex dynamic patterns in time series is of both scientific and practical significance. The development of robust and effective forecasting models remains a pressing need, especially as the volume and complexity of time series data continue to grow in the era of big data and the Internet of Things.

In recent years, deep learning has brought major progress to time series forecasting. Researchers have developed several types of models for this task, including those based on convolutional neural networks (CNNs) [9–11], recurrent neural networks (RNNs) [12–14], Transformer models [15–19], and multilayer perceptrons (MLPs) [8,20–22]. Each architecture leverages distinct mechanisms to capture temporal patterns, dependencies, and multi-scale features inherent in time series data. Most current methods follow two main strategie: sequence decomposition and multi-period analysis. Sequence decomposition is a traditional approach in time series research. For example, the Autoformer model uses this method, as proposed by Wu et al [17]. It decomposes complex signals into more predictable components such as seasonal and trend components, thereby simplifying the modeling task. The latter splits mixed temporal variations into multiple components with different periodic lengths, enabling the model to capture intrinsic properties and maintain the regularity of signals, thus improving forecasting accuracy. Recently, MLP-based models have demonstrated the potential to outperform Transformer variants in multivariate time series forecasting, attracting increasing attention from the research community. For instance, the TimeMixer [8] model and the TSMixer model both use MLPs to mix information from different sources. TSMixer [21] fuses data in both the temporal and channel domains. This design helps the model learn dependencies across dimensions. However, TSMixer needs a long look-back window. This requirement increases computational cost and limits its use in large-scale or real-time tasks. TimeMixer takes a different approach. It decomposes sequences into seasonal and trend components, then mixes them across scales. This method improves efficiency and achieves strong results on many benchmarks. Despite these advances, several problems remain. Simple decomposition often misses key details. Mixing across scales can lead to information loss [20]. Real-world time series often show sudden spikes and drops. Traditional methods struggle to explain these changes. Models that focus only on time may overlook important relationships. Nonlinear patterns, like sharp peaks or hidden cycles, are also difficult to capture. These challenges highlight the need for better models. Future approaches should use both temporal and frequency information. They should also combine features from different scales. Modeling complex time series accurately is still difficult. This challenge motivates the search for new hybrid models.

To overcome the limitations identified above, we propose **STF-DKANMixer**, a hybrid model that combines multilayer perceptrons (MLPs) with Kolmogorov–Arnold Networks (KANs) [23]. STF-DKANMixer leverages Fourier transforms to generate multi-scale representations of temporal dynamics. In the Past-Information-Mixing (PIM) stage, the input sequence is decomposed into seasonal (S), trend (*τ*), and frequency (F) components. This tri-component decomposition enables the model to capture both regular and irregular patterns, including the abrupt changes frequently observed in real data. Each component is processed by the **GELUKAN** module, which extracts short-term fluctuations, long-term trends, and nonlinear relationships in both the time and frequency domains. The **deformable-attention (DFA) block** then adaptively fuses information across scales [24], enhancing the model’s flexibility and robustness when handling multivariate time series. During inference, the Future-Information-Mixing (FIM) module aggregates predictions from earlier steps to further improve accuracy. Thanks to this architecture, STF-DKANMixer consistently outperforms existing methods in long-sequence forecasting while maintaining a compact computational footprint.

The main contributions and innovations of this paper are as follows:

(1) We fully leverage and improve the characteristics of KAN [23], ingeniously combining KAN with MLP. This enables the model to maintain the simplicity and efficiency of the MLP structure while utilizing the deep nonlinear extraction capability of KAN, thereby alleviating the catastrophic forgetting problem of MLP and more accurately capturing subtle dynamic changes in time series data.

(2) We propose an innovative deformable attention module that dynamically and adaptively samples key features at each scale, effectively capturing nonlinear relationships and sudden changes in time series data. Unlike traditional fixed sampling methods, our improved mechanism not only performs well in the computer vision domain but also fully exploits multi-scale information in time series forecasting, thereby enhancing overall predictive performance.

(3) We introduce a novel model composed of three core components. The **DFT-based tri-series decomposition module extracts trend and seasonal components through Discrete Fourier Transform (DFT), while the residual is further refined into high-frequency details via a Haar wavelet transform with error compensation. Building on this decomposition, the GELUKAN network captures local nonlinear dynamics, and the proposed DFA module integrates global temporal dependencies, jointly enhancing the model’s ability to represent complex temporal variations.**

(4) Our model, STF-DKANMixer, achieves state-of-the-art performance in long-term forecasting on multiple public benchmark datasets, demonstrating its effectiveness and generalizability.

The remainder of this paper is organized as follows: Sect 2 reviews related work, Sect 3 details the model structure and key modules, Sect 4 presents experimental design and result analysis, and Sect 5 concludes the paper and discusses future work. Through these contributions, we aim to advance the state of the art in time series forecasting and provide a solid foundation for future research in this area.

## 2 Related work

Recent years have witnessed remarkable progress in the field of time series forecasting, fueled by the proliferation of large-scale sequential datasets and the rapid evolution of deep learning techniques. Time series forecasting has become a cornerstone in a wide array of application domains, including but not limited to finance, energy management, transportation, healthcare, and meteorology. Despite these advances, accurately modeling and predicting real-world time series remains a formidable challenge due to the inherent complexity, non-stationarity, and the presence of diverse temporal patterns such as trends, seasonality, abrupt changes, and noise. Traditional statistical methods, while effective for stationary and linear processes, often fall short when confronted with the nonlinear and multi-scale characteristics of modern time series data.

To address these challenges, the research community has developed a rich variety of approaches that leverage the representational power of deep neural networks. Early efforts focused on recurrent neural networks (RNNs) and their variants, which are capable of capturing sequential dependencies but often suffer from issues such as vanishing gradients and limited long-term memory. Convolutional neural networks (CNNs) were subsequently introduced to model local temporal patterns and improve computational efficiency, yet their receptive fields are inherently limited. The advent of Transformer-based architectures marked a significant breakthrough, as self-attention mechanisms enable the modeling of long-range dependencies and complex interactions within the data. More recently, multilayer perceptron (MLP)-based models have gained traction for their simplicity, scalability, and competitive performance, especially when equipped with advanced mixing and decomposition strategies.

In parallel with architectural innovations, researchers have increasingly recognized the importance of multi-scale and multi-frequency analysis in time series forecasting. Techniques such as seasonal-trend decomposition, wavelet transforms, and Fourier analysis have been integrated into deep learning frameworks to disentangle and exploit the various components underlying observed sequences. Furthermore, hybrid models that combine different neural network paradigms—such as RNNs, CNNs, Transformers, and MLPs—have been proposed to harness their complementary strengths and overcome the limitations of individual approaches. Despite these advancements, several open problems remain, including the effective fusion of multi-scale features, the modeling of nonlinear relationships, and the robust handling of non-stationary and noisy data.

In the following subsections, we provide a comprehensive review of the most influential research directions in this field, including time mixing and deep learning models for time series forecasting, the development and application of KANs, and advanced time decomposition methods that underpin state-of-the-art forecasting performance.

### 2.1 Time mixing and time series forecasting

Time series forecasting is a fundamental task that leverages past observations to predict future values, playing a critical role in domains such as traffic planning and weather forecasting. In real–world applications, however, the underlying sequences often exhibit complex, non-stationary behaviors—including gradual uptrends, downtrends, and abrupt fluctuations—which pose significant challenges for accurate prediction. These intertwined temporal patterns can undermine models that assume smooth or linear dynamics, necessitating advanced techniques capable of disentangling multi-scale signals and capturing both low-frequency trends and high-frequency anomalies for robust forecasting performance.

Accurately modeling temporal dependencies in time series data remains one of the field’s most pressing challenges, driving extensive research into specialized deep-learning architectures. Broadly speaking, these solutions can be classified into four main categories according to their core building blocks: recurrent neural network (RNN)–based, convolutional neural network (CNN)–based, Transformer-based, and multilayer perceptron (MLP)–based methods. CNN-based models—such as the Temporal Convolutional Network (TCN) and Informer—apply one-dimensional convolutional filters along the temporal axis to capture localized patterns. They often employ dilated or stacked convolutions to extend their receptive fields, yet they may still struggle to learn dependencies spanning very long horizons. RNN-based approaches, exemplified by the LSTNet framework introduced in 2018 and the gated recurrence design proposed in 2017, use recursive hidden-state updates to propagate information sequentially through time. While RNNs inherently maintain memory of past inputs, their sequential nature and finite state capacity limit their ability to retain information over extended sequences. More recently, Transformer-derived architectures like Informer and Autoformer have achieved broad acclaim for long-horizon forecasting by leveraging self-attention layers that adaptively weight the relevance of each past time step when predicting future values. Parallel to this, purely MLP-based designs have been introduced to time series forecasting; by employing fully connected mixing layers, these models blend information across both time and feature dimensions in a single operation, yielding competitive accuracy alongside significant runtime and parameter efficiency.

With the increasing demand for time series prediction, a single deep model structure is not enough. More and more deep models have begun to have multi-scale mixed structures, such as Pyraformer, Cone Attention, and SCINet, TimeMixer. However, some of these models do not utilize different scale information extracted from past observations for future predictions, and some do not pay attention to nonlinear relationships in the sequence, resulting in insufficient extraction of peak points.

Different from the above model, this paper not only studies the multi-scale hybrid structure in time series prediction, but also studies the nonlinear relationship on this basis. In STF-DKANMixer, we propose a new multi-scale hybrid architecture based on KAN and MLP. We use GELUKAN to improve the ability of multi-scale hybrid structure to capture nonlinear relationships, use DFA blocks to improve the accuracy of feature fusion, and DFT (Fourier decomposition) to make up for the lack of multi variable ability of average pooling decomposition, so as to improve the performance of the model.

### 2.2 Kolmogorov-Arnold network

The Kolmogorov–Arnold representation theorem establishes that any continuous function of multiple variables can be exactly expressed as a finite sum of continuous univariate functions composed with addition. Building on this mathematical result, the Kolmogorov–Arnold Network (KAN) was introduced as an alternative to the standard multilayer perceptron (MLP) architecture. Unlike conventional MLPs, which apply fixed activation functions at each neuron, KAN replaces each scalar weight with a learnable one-dimensional activation function positioned along the connection between neurons. Since its inception and growing recognition, a variety of KAN variants have emerged, reflecting the network’s versatility and flexibility; as a result, KAN is increasingly regarded as a promising substitute for traditional MLPs [22].

In our view, KAN and MLP architectures are not mutually exclusive choices but can complement one another within a unified model. To demonstrate this, we propose STF-DKANMixer, a hybrid framework that interleaves KAN and MLP components to leverage the strengths of both. Notably, similar hybrid approaches have begun to appear in recent literature—for example, TSKANMixer integrates KAN layers into the TSMixer architecture to improve its capacity for learning complex nonlinear relationships [23]. While TSKANMixer focuses primarily on enhancing the representation of nonlinear interactions among decomposed variables [24], our STF-DKANMixer goes further by embedding a specialized GELUKAN module designed to extract and model nonlinear dynamics more effectively across multiple scales.

Specifically, STF-DKANMixer uses GELUKAN units at various fusion points to capture subtle nonlinear dependencies that may be overlooked by either pure MLP or pure KAN designs alone. At the same time, the MLP portions of the network ensure efficient global information mixing and maintain a compact parameter footprint. This interleaving strategy not only preserves the computational simplicity of MLPs but also harnesses the deep approximation power of KANs, mitigating issues such as catastrophic forgetting and enabling more precise modeling of intricate time-series behaviors. By combining these complementary mechanisms, STF-DKANMixer achieves a balance between expressiveness and efficiency, making it a robust choice for complex forecasting tasks.

### 2.3 Time decomposition

In order to make full use of the various potential patterns in the time series in the real world and the characteristics of different patterns, methods in recent years have tried to divide various time series into multiple sub components. The common ones are: trend seasonal decomposition, multi-scale decomposition, multi period decomposition, and multi frequency decomposition. For example, timemixer decomposes the sequence according to the principle from coarse to fine to form multi-scale variables with different time dimensions across domains. PDF and timesnet use Fourier cycle analysis to decompose the time series into multiple sub cycle sequences based on the calculated period. Dliner selects the moving average to separate the seasonal and trend components in the sequence. Scinet uses the hierarchical down sampling tree to extract and exchange information iteratively from multiple time resolutions. Inspired by this, this paper proposes a new mixed structure of MLP and KAN to decompose the variables of season, trend and frequency. This structure not only considers the direction of season and trend, but also considers the multi frequency domain. Decompose and analyze time series to accurately model the complex patterns in the world series and solve the problem of difficult domain modeling.

## 3 Methodology

Time series forecasting aims to predict future values of a sequence based on its historical observations. Formally, given a historical univariate or multivariate time series can be shown as $x\in \;{\mathbb{R}}^{N\times P}$, where *N* denotes the number of variables and *P* is the length of the input window, the objective is to forecast the future sequence $x_{L}\in \;R^{N\times F}$ over a prediction window of length *F*. This task is fundamental in a wide range of real-world applications, including finance, energy, transportation, and meteorology, where accurate forecasting can drive critical decision-making. However, real-world time series data are often not only highly complex, exhibiting non-stationary behaviors, intricate temporal dependencies, but also a mixture of patterns such as trends, seasonality, and abrupt fluctuations. These characteristics pose significant challenges for traditional forecasting models, which may struggle to disentangle and capture the multi-scale and nonlinear dynamics inherent in the data. Analogous to the “trans-spectral” phenomena observed in physical media—where energy is redistributed across different scales and frequency bands [25]—we advocate for modeling frameworks capable of scale-aware decomposition, representation, and prediction. Therefore, there is a pressing need for advanced modeling frameworks that can effectively decompose, represent, and predict such complex temporal patterns, enabling robust and accurate time series forecasting in practical scenarios.

### 3.1 Overall architecture

**Fig 1:**
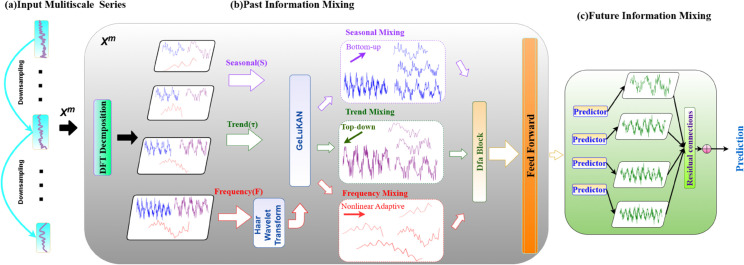
The architecture of STF-DKANMixer framework, consisting of (a) Input Multiscale Series, (b) Past Information Mixing, and (c) Future Information Mixing components.

Given a historical univariate or multivariate input time series as $x\in \;{\mathbb{R}}^{N\times P}$, where *N* is the number of variables and *P* is the input window length, the goal of time series forecasting is to predict the future sequence $x_{L}\in \;{\mathbb{R}}^{N\times F}$ over a window of length *F*. To address the inherent complexity and multi-scale characteristics of real-world time series, we propose the STF-DKANMixer framework, whose overall structure is illustrated in Fig 1. As shown in Fig 1, the STF-DKANMixer first decomposes the input sequence into multiple components, including seasonal, trend, and frequency components, to disentangle different temporal patterns. These components are then processed through a multi-scale hybrid backbone that integrates the complementary strengths of Kolmogorov-Arnold Networks (KAN) and multilayer perceptrons (MLP). A key innovation of our model is the deformable attention (DFA) mechanism, which adaptively fuses features across different scales and components [26], thereby enhancing the extraction of nonlinear and long-range dependencies. Specifically, the Past-Information-Mixing (PIM) module is responsible for extracting rich historical features from the decomposed sequences, while the Future-Information-Mixing (FIM) module aggregates these features to generate the accurate future predictions. This holistic design enables STF-DKANMixer to robustly capture both global trends and local fluctuations, that providing a powerful and flexible solution for complex time series forecasting tasks. The following sections provide very detailed descriptions of each module within the STF-DKANMixer framework.

### 3.2 Multiscale mixing KAN And Mlp

In time series analysis, classical multi-scale decomposition frameworks exploit natural differences across resolutions by capturing fine-grained details (e.g., minute-level trading fluctuations) at small scales and modeling macro trends (e.g., quarterly economic cycles) at coarse scales. However, this fixed-resolution paradigm has two major limitations. First, modules such as the PDM module in TimeMixer rigidly partition scales via predefined downsampling rates (such as halving the sequence length at each layer), failing to adapt to dynamic frequency changes observed in real-world scenarios (e.g., abrupt spectral shifts in traffic flow under extreme weather). Second, although AutoFormer separates seasonal and trend components using a decomposition strategy, it still overlooks the dynamic interactions between frequency components and other temporal factors; existing methods lack effective mechanisms to model such cross-frequency interdependencies in time series. To address these issues, we propose STF-DKANMixer which is a hybrid MLP–KAN architecture built upon a multi-scale mixing backbone. This design leverages the strengths of traditional seasonal–trend decomposition, introduces dynamic frequency components through the MLP–KAN combination, and applies distinct modules for past feature extraction and future prediction stages.

As shown in Fig 1, in order to cope with complex changes, we first construct multi-scale representations through hierarchical downsampling of the input sequence $x\in {\mathbb{R}}^{P\times V}$, obtaining $X=\{X_{0},\ldots ,X_{I}\}$, where $X_{i}\in {\mathbb{R}}^{\left[\frac{P}{2^{i}}\right]\times C}$ and i∈{0,…,I}, *V* is the number of variables. Among them, the sequence of the lowest scale is *x*_0_ = *x*, which is the input sequence, representing the most subtle time changes in the sequence, while the sequence of the highest scale *x*_*I*_ is specifically for the macro changes in the sequence. Subsequently, within each scale, we apply our DFT-based tri-component decomposition to extract seasonal, trend, and frequency components, enabling comprehensive capture of multi-frequency temporal patterns. The multi-scale sequences are then mapped into the deep feature space *X*^0^ via the embedding layer, expressed as *X*^0^ = *Embed*(*X*). By using this design, we can obtain a multi-scale representation of the input sequence with rich decomposed features.

After that, we can use stacked past-information-mixing block (PIM) to mix all the decomposed input sequences of different scales. For layer m, the input is *X^m−1^*, and the operation process of PIM can be expressed in Eq 1:

$$X^{\;m}=PIM(X^{m-1}),m\in \{0,\ldots ,L\}$$ (1)

Where M is the total number of layers of PIM, $X^{\;m}=\{x_{0}^{l},\ldots ,x_{I}^{l}\}$, $x_{i}^{l}\in {\mathbb{R}}^{[\frac{P}{2^{i}}]\times d_{model}}$ denotes the mixed past representations with *d*_*model*_ channels. The next section will provide a detailed description of the PIM module.

In the prediction phase for future information, we utilize the Future Information Mixture (FIM) block to integrate the extracted multi-scale historical information *x^M^* and produce future forecasts, as illustrated below in Eq 2:

$$\hat{x}=FIM(X^{\;m})$$ (2)

Where $\hat{x}\in {\mathbb{R}}^{F\times C}$ is the result of the final prediction. Through the above design, our model STF-DKANMixer can finally obtain important past information from decoupled multi-scale observations, and use the combination of frequency domain information, nonlinear information and multi-scale past information to predict the future.

### 3.3 Past-Information-Mixing (PIM)

Real-world time series are influenced by many factors and often exhibit complex, mixed dynamics across scales, we can observe that even on the coarsest scale, the historical observation data also show multiple changes including seasonal, trend and frequency components. As shown in Fig 1, the coarsest-scale sequence exhibits clear seasonality and trend as well as rich frequency-domain content. These components have their own unique properties in time series analysis [27], which correspond to short-term, medium-term and long-term dynamic changes, or reflect different characteristics of steady-state and unsteady-state. Based on this causal relationship, we propose the “past information mixing (PIM) block. Its design concept is to break through the limitations of the traditional unified processing of multi-scale sequences, and to achieve a more detailed and efficient modeling of multi-scale time series dynamics through decomposing multi scale and fusion of seasonal, trend and frequency components respectively, supplemented by KAN network to capture the complex nonlinear characteristics in the sequence.

#### 3.3.1 DFT-based decomposition framework.

To address the limitations of traditional moving average decomposition methods, our approach employs an enhanced Discrete Fourier Transform (DFT) framework for precise seasonal-trend separation. Unlike conventional approaches that suffer from spectral leakage and boundary effects, our method leverages the superior frequency selectivity of DFT analysis to achieve more accurate component isolation.

**Frequency domain analysis:** The decomposition begins by transforming time-domain signals into the frequency domain using real-valued Fast Fourier Transform operations. This transformation enables direct manipulation of spectral components, allowing for precise identification and extraction of periodic patterns. The DC component is systematically removed to eliminate constant bias effects that could interfere with subsequent seasonal pattern identification.

**Adaptive spectral filtering:** Rather than employing fixed frequency bands, our methodology implements an energy-based selection strategy that identifies the most significant spectral components. Through ranking frequency magnitudes and retaining only the dominant components, the approach effectively separates meaningful periodic signals from noise. This adaptive filtering mechanism ensures that seasonal reconstruction focuses on the most informative frequency content while discarding spurious oscillations.

**Component reconstruction strategy:** The seasonal component is reconstructed through inverse Fourier transformation of the filtered frequency domain representation, ensuring that only the most significant cyclical behaviors are preserved. The trend component is subsequently obtained as the residual between the original signal and the reconstructed seasonal component, naturally capturing long-term directional changes and smooth variations that lack periodic characteristics.

This DFT-based decomposition framework provides several theoretical advantages over traditional methods: (1) enhanced frequency resolution through direct spectral manipulation, (2) elimination of phase distortion commonly introduced by moving average filters, (3) preservation of temporal causality through careful boundary handling, and (4) adaptive frequency selection that accommodates varying spectral characteristics across different datasets.

Specifically, for the first PIM block, we first use the multi-scale sequence where the idea inspired by decomposition module from timemixer [8], but we enrich the scale of input. The input sequence *x*_*M*_is decomposed into seasonal components $S^{\;m}=\{s_{0}^{\;m},\ldots ,s_{I}^{\;m}\}$ and trend components $\tau^{m}=\{t_{0}^{\;m},\ldots ,t_{I}^{\;m}\}$. Considering that the seasonal and trend components reflect the dynamic characteristics of short-term and long-term changes in the sequence respectively [28], We further obtain the frequency component $F^{\;m}=\{f_{0}^{\;m},\ldots ,f_{I}^{\;m}\}$ as the residual after removing seasonal and trend components from the original sequence. This frequency variable not only captures the high-frequency instantaneous fluctuation that is easy to be ignored in the traditional decomposition method, but also reflects the mode of local unsteady changes in the data [29]. By mixing frequency variables with seasonal and trend components in parallel on multiple scales, we can obtain a more comprehensive and refined multi-scale time series feature representation. In short, the m-th PIM block can be formalized as in Eq 3 :

$$s_{i}^{\;m},t_{i}^{\;m},f_{i}^{\;m}=TSeriesDecomp(x_{i}^{\;m}),i\in \{0,\ldots ,I\}\;x^{\;m}=x^{m-1}+FeedForward(S-Mix(g(\{s_{i}^{\;m}{\}}_{i=0}^{I}))\;\;+T-Mix(g(\{t_{i}^{\;m}{\}}_{i=0}^{I}))\;\;+F-Mix(g(\{f_{i}^{\;m}{\}}_{i=0}^{I})))$$ (3)

Among them, the feedforward layer (⋅) has two linear layers, with the intermediate activation function GELU for information interaction between channels. S-mix (⋅), T-mix (⋅) and F-mix (⋅) represent seasonal mixing, trend mixing and frequency mixing. G (⋅) in each mix represents the GELUKAN block we designed to capture nonlinear information within each scale of the decomposed component. The next section we will introduce GELUKAN in detail.

### 3.4 GELUKAN nonlinear information

After decomposition, we get the seasonal component $S^{\;m}=\{s_{0}^{\;m},\ldots ,s_{I}^{\;m}\}$, the trend component $\tau^{m}=\{t_{0}^{\;m},\ldots ,t_{I}^{\;m}\}$ and the frequency component $F^{\;m}=\{f_{0}^{\;m},\ldots ,f_{I}^{\;m}\}$ respectively. In order to get the nonlinear relationship more accurately, we propose the GELUKAN module to realize the long-term modeling of nonlinear pattern extraction and time-dependent learning in the sequence. GELUKAN inherits the properties of ReLUKAN, who is good at solving differential equations, and replaces the double ReLU activation function [30] with the GELU activation function and defines a new basis function. Our work is inspired by the idea of HRkANs [31], and the basis function we choose is GELU. This design enables efficient collaboration between local feature extraction and global dynamic fusion, thereby improving the performance of the model’s accuracy and robustness. Specifically, The GELUKAN module uses a smooth GELU activation instead of traditional ReLU, which not only overcomes the discontinuity of activation function at the boundary, but also realizes the smooth transfer of gradient in the process of nonlinear mapping, thus ensuring the effective fusion of information at all scales. The basis function can be formulated as in Eq 4 :

$$G_{m,i}(𝐱)=\text{GELU}(e_{i}-𝐱{)}^{\;m}\times \text{GELU}(𝐱-s_{i}{)}^{\;m}\times R_{m}$$ (4)

where *m* is the current number of layers,*e*_*i*_ and *s*_*i*_, represent the upper and lower bounds of the basis function respectively, $R_{m}\;=\;(\frac{2}{e_{i}-s_{i}}{)}^{2m}$ is the normalized constant. GELUKAN is applied to the processing of all scales of decomposed components, which is not limited to specific scales. This design can enable our model to capture nonlinear relationships on different time scales. Specifically, for the seasonal component, trend component and frequency component of each scale, we use GELUKAN for nonlinear transformation:

- for seasonal components, GELUKAN can capture complex cyclical patterns
- for trend components, GELUKAN can model long-term dependence and rate of change
- for frequency components, GELUKAN can extract high-frequency detail features in the signal

**Fig 2:**
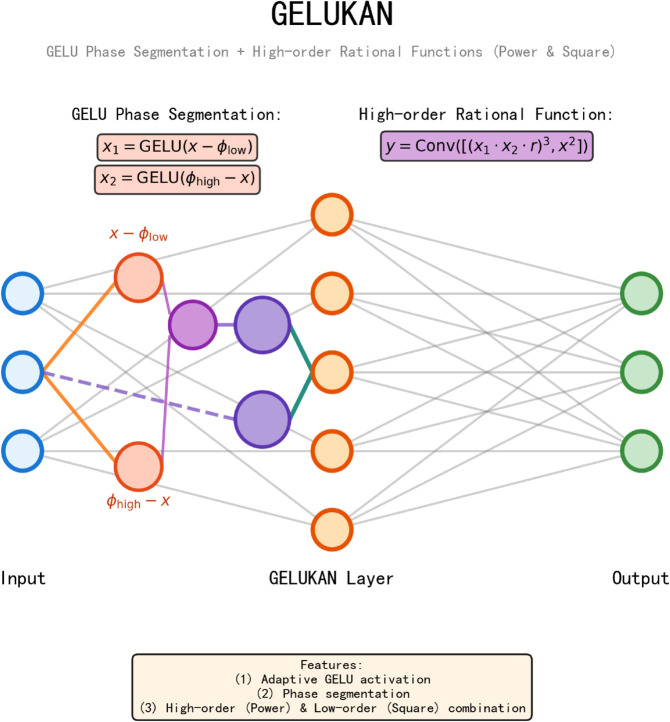
The architecture of GELUKAN.

In this way, GELUKAN is applied to all scales and components. Therefore, after we introduce GELUKAN module to all components and scales, our model can systematically learn and express the nonlinear characteristics of various variables after time series decomposition, thus significantly improving the prediction performance and robustness. In addition, the interpretability of GELUKAN module provides an intuitive perspective for in-depth analysis of the internal structure of time series, helps to understand the internal dynamics and potential laws in the data, and lays a solid foundation for subsequent theoretical analysis and application optimization (Fig 2).

### 3.5 Seasonal mixing

Box and Jenkins’ research in 1970 found that in seasonal analysis, the longer time period is actually the summary of the shorter time period data. For example, aggregating seven days’ daily traffic flow data into one week’s data highlights the key role of detailed information in predicting seasonal trends [32].

Therefore, in the seasonal mixed analysis, we adopted a fine to coarse analysis strategy, that is, the detailed time series data on the lower time scale were summarized to a higher level. Through this method, the detailed information can be supplemented by the seasonal modeling on the coarser scale in the series. Technically, for a group of multi-scale seasonal components $S^{\;m}=\{s_{0}^{\;m},\ldots ,s_{I}^{\;m}\}$, We use the residual connection strategy to realize the interaction of bottom-up seasonal information through the bottom-up mixing layer of the m-th scale. This process can be formally expressed as in Eq 5 :

$$\text{for i : 1}\to I\text{ do: }s_{i}^{\;m}=s_{i}^{\;m}+BUM(s_{i-1}^{\;m})$$ (5)

**Fig 3:**
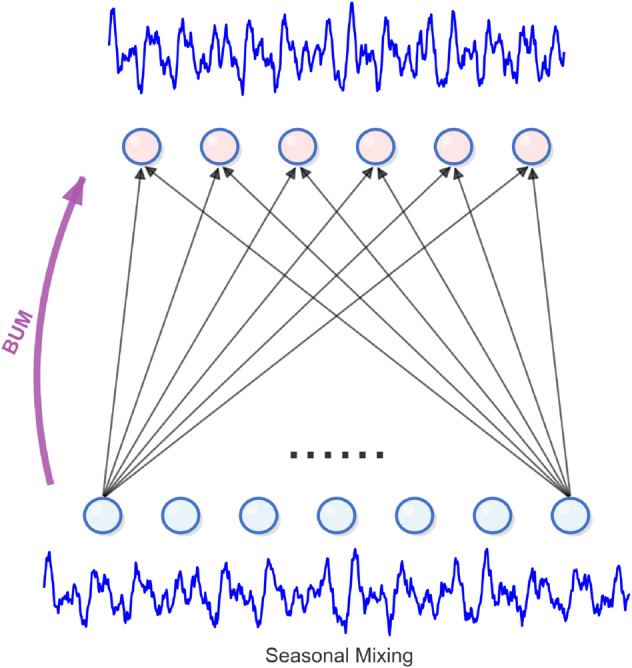
The temporal linear layer in seasonal mixing.

Where bum (.) represents bottom-up mixing, which is composed of two linear layers arranged in sequence, and the GELU activation function of time is inserted in the time dimension. The design can gradually capture and transfer the local time pattern, realize the bottom-up efficient fusion of seasonal characteristics, and significantly increase the expression ability of the model for complex time-varying dynamics. The input dimension is $\left\lfloor \frac{P}{2^{i-1}}\right\rfloor$ and the output dimension is $\left\lfloor \frac{p}{2^{i}}\right\rfloor$, we can see Fig 3 for details.

### 3.6 Trend mixing

Compared with the seasonal component, the detail fluctuation in the trend item is easier to introduce interference in refining the macro trend. It is worth mentioning that the upper level time series with coarser granularity can more intuitively reflect the overall trend than those with finer granularity. Based on this, we adopt a top-down hybrid strategy, and use the macro information provided by the coarse scale to guide the finer scale trend modeling.

In multi-level trend modeling, as for multi-scale trend component we showed in previous $\tau^{m}=\{t_{0}^{\;m},\ldots ,t_{I}^{\;m}\}$, We build a cross scale trend interaction system through the residual correction mechanism: the TDM layer is designed for the i-th scale to realize the directional migration from coarse-grained trend features to fine-grained features, specifically using a top-down hierarchical modeling architecture in Eq 6:

$$\text{for i : (I - 1)}\to 0\text{ do: }\tau_{i}^{\;m}=\tau_{i}^{\;m}+TDM(\tau_{i+1}^{\;m})$$ (6)

**Fig 4:**
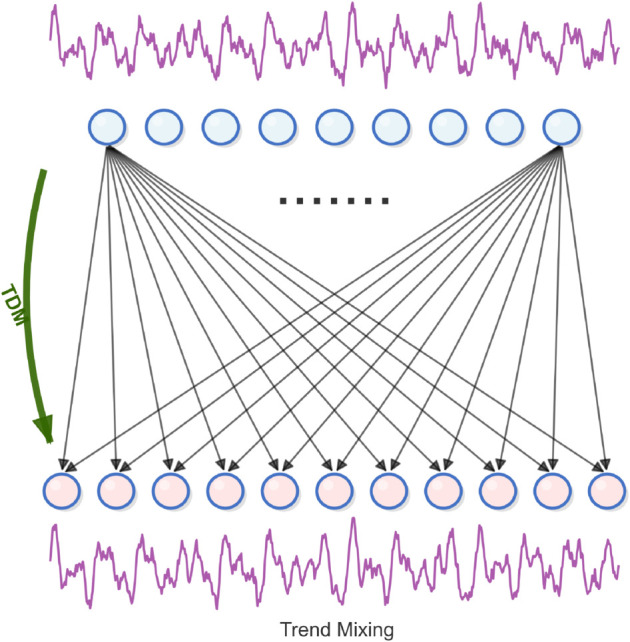
The temporal linear layer in trend mixing.

Where TDM (.) refers to self-determined downward mixing, which is composed of two linear layers arranged in sequence, and the GELU activation function of time is inserted in the time dimension. The input dimension is $\left\lfloor \frac{P}{2^{i+1}}\right\rfloor$ and the output is $\left\lfloor \frac{p}{2^{i}}\right\rfloor$, we can see Fig 4 for details.

### 3.7 Frequency mixing

The frequency component captures the characteristics of high-frequency fluctuations and short-term fluctuations in the time series, which are neither seasonal nor long-term trends. In order to effectively process these high-frequency signals, we designed a special frequency mixing module to extract and enhance these subtle fluctuations that may contain important prediction information.

AS same as the mixed processing of seasonal and trend components, we also use multi-scale processing strategy for frequency components. For multi-scale frequency component $F^{\;m}=\{f_{0}^{\;m},\ldots ,f_{I}^{\;m}\}$. Therefore, we introduce a nonlinear adaptive hybrid mechanism.

We first obtain the original frequency component by subtracting the seasonal component and trend component in the sequence, which can be expressed as follows in Eq 7:

$$fo_{i}=x_{i}-s_{i}-\tau_{i}$$ (7)

Where *x*_*i*_ is the original time series, *fo*_*i*_ is the raw frequency component,*s*_*i*_ is the seasonal component and $\tau_{i}$ is the trend component.

We employ a GPU-accelerated Haar wavelet decomposition to extract frequency components [33]. Directly using the raw residual mixes global variations with local noise, leading to spectral leakage and noise amplification that hinder the learning of periodic features. In contrast to approaches that retain only high-frequency details [34], our method fuses both low-frequency (approximation) and high-frequency (detail) bands, thereby fully capturing both global and local characteristics of the signal. The use of fixed convolutional kernels enables parallel Haar transforms on the GPU, substantially accelerating both training and inference. The process can be expressed as follows in Eq 8:

$${cA}_{i}=\frac{1}{\sqrt{2}}\left(fo_{2i}+fo_{2i+1}\right)\;\;{cD}_{i}=\frac{1}{\sqrt{2}}\left(fo_{2i}-fo_{2i+1}\right)$$ (8)

Where **cA** denotes the approximation (low-frequency) coefficients, **cD** denotes the detail (high-frequency) coefficients. To match the original sequence length, we upsample **cA** and **cD** to length *P* via linear interpolation, and fuse them as in Eq 9:

f=Upsample(cA)+Upsample(cD) (9)

Our Haar wavelet decomposition ensures temporal causality through careful mathematical design. The decomposition employs stride-2 convolution operations with Haar filters $\left[1/\sqrt{2},1/\sqrt{2}\right]$ (low-frequency) and $\left[1/\sqrt{2},-1/\sqrt{2}\right]$ (high-frequency) to process consecutive pairs of historical points. This approach naturally produces approximation coefficients **cA** and detail coefficients **cD** of length ⌊P/2⌋ using exclusively historical observations. At the sequence boundaries, coefficients are computed strictly within the available historical window without extrapolation, ensuring no artificial padding or future information is introduced. For sequence reconstruction, we employ linear interpolation to upsample both coefficient sequences back to the original length *P*. The interpolation process maintains temporal alignment while using only the computed historical coefficients, thereby preventing future information contamination. The final frequency component f=Upsample(cA)+Upsample(cD) captures comprehensive spectral characteristics while preserving strict temporal causality, as each output element depends solely on historical observations within the input window [1,*P*]. In summary, our design (i) avoids artificial padding at boundaries, (ii) ensures stride-2 alignment of sampling pairs, and (iii) reconstructs sequences solely from historical coefficients, thereby fully eliminating potential data leakage.

Then, for each scale I, we use GLUKAN to carry out nonlinear transformation to process the obtained initial frequency component in Eq 10:

$$\text{for i : 1}\to I\text{ do: }f1_{i}^{\;m}=GELUKAN(f_{i}^{\;m})$$ (10)

Where GELUKAN (⋅) refers to the nonlinear converter we use, which can capture the complex nonlinear relationship in the frequency component. We especially choose KAN implementation based on GELU activation function, because the smoothing properties of GELU function are more suitable for processing subtle changes in frequency signals. *f*1 (⋅) represents the frequency component processed by GELUKAN.

Of course, there is no doubt that each scale has different weights in the sequence, so we introduce adaptive weights to better simulate the frequency transformation in the sequence, which can be expressed by the following formula in Eq 11:

$$\omega_{i}=\sigma (\phi (f1_{i}^{\;m})),\;fw_{i}^{w}=\omega_{i}\times f1_{i}^{\;m}$$ (11)

**Fig 5:**
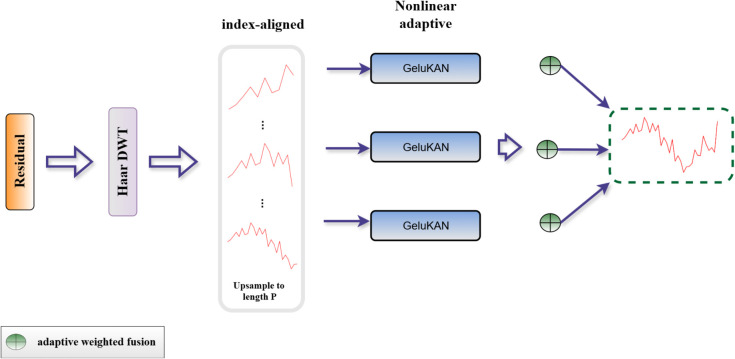
The architecture of frequency mixing.

The normalization function *σ*(⋅), weight computation network *ϕ*(⋅), and learned weighted frequency components *fw*(⋅) form a differentiable attention mechanism that automatically discriminates between meaningful spectral patterns and irrelevant noise through gradient-based optimization. This frequency-aware architecture (illustrated as ⊕ in Fig 5) enables adaptive signal enhancement by dynamically adjusting spectral emphasis during forward propagation.

Finally, we fuse frequency components of different scales, which can be expressed as follows in Eq 12:

$$f_{i}^{\;m}=\sum_{j=0}^{I}fw_{j}^{w}$$ (12)

Where *j* denotes the summation index over all scales. This design clearly expresses that each scale frequency component has its own special GELUKAN processing module. GELUKAN is used to enhance the nonlinear characteristics in the frequency component. The processed frequency components are integrated through the adaptive fusion mechanism, which is significantly different from the top-down mixing of trend components and the bottom-up mixing of seasonal components, highlighting the parallel processing characteristics of frequency mixing. See Fig 5 for details.

### 3.8 DFA block

In time series prediction, effectively capturing multi-scale features and long-distance dependence is the key challenge to improve the performance of the model. Although the input sequence can be divided into three components: seasonal, trend and frequency to capture different time patterns, the interaction and integration between the three still need to be handled more carefully. Therefore, we designed DFA block to process the fused components in depth. The module is based on the deformable attention mechanism and supplemented by the adaptive weight adjustment strategy, which significantly enhances the ability of the model to fuse multi-scale features. Deformable attention mechanism was first introduced in the field of computer vision to flexibly adjust the sampling position on the feature map to more effectively capture the local details and global context in the image. We introduce this innovative mechanism into the field of time series and develop an enhanced DFA module. By applying deformable attention on the time series, the DFA module can adaptively adjust the sampling position and capture the key patterns and multi-scale features in the time series. This cross domain application not only improves the prediction accuracy of the model, but also provides a new perspective for the modeling of complex time series data.

The DFA module is designed to enhance the interaction and fusion capabilities of decomposed temporal components across multiple scales. To achieve this, we incorporate several mechanisms that simultaneously improve flexibility and stability. First, features from different resolutions are aligned through interpolation to ensure temporal dimension consistency. Subsequently, reference points are generated through regular grids and corrected with learnable offsets, enabling adaptive multi-point sampling to better locate key temporal patterns.

The attention computation is implemented based on a deformable attention structure adapted from RT-DETR, specifically tailored for time series tasks. Combined with a multi-level feature pyramid, this module can aggregate information across different temporal resolutions, balancing local fine-grained variations with global long-term dependencies. To ensure training robustness, normalization and stability control mechanisms are introduced in the attention computation, while a lightweight residual refinement strategy achieves balance between aggregated features and original queries.

Through this design, DFA provides a stable yet highly expressive multi-scale fusion mechanism, enabling STF-DKANMixer to more effectively capture nonlinear dependencies and sudden changes in complex time series.

The core idea of DFA module is to adaptively sample feature maps of different scales by learning the offset of reference points. Specifically, DFA generates *q* generate initial reference point $p_{ref}=sigmoid(\phi_{ref}(q))$, and the offset $\Delta p=\phi_{offset}(q)$ we can obtained it through network learning, then we adjust the sampling position *p* = *p*_*ref*_  +  Δp. This design enables the model to focus on the most relevant time points and scales. In the DFA block, the attention weight *ω* not only depends on the similarity of query and key,but also combines the importance weight *α* of scale. According to the formula, $\omega \;=\;\text{softmax}(\phi_{\text{weight}}(q)\cdot \alpha )$ is obtained. In this way, DFA can dynamically adjust the attention allocation according to the feature importance of different scales, so as to achieve more accurate feature aggregation.

**Fig 6:**
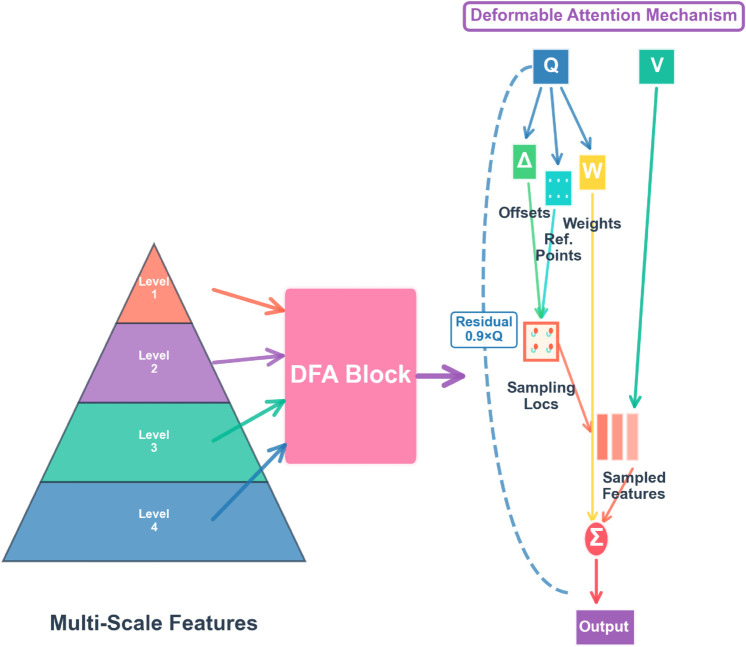
The Structure of DFA Blocks.

By introducing the DFA module, STF-DKANMixer has not only significantly improved the prediction accuracy, but also showed stronger robustness and flexibility when processing complex time series data. Experimental results show that DFA can effectively capture multi-scale features and long-distance dependence, and provide a more powerful modeling ability for time series prediction. We can See Fig 6 for details.

### 3.9 Future-Information-Mixing (FIM)

After passing through M PIM blocks, we obtain multi-scale historical information, expressed as $X^{M}=\{x_{0}^{M},\cdots ,X_{I}^{M}\}$, where $x_{i}^{M}\in {\mathbb{R}}^{\frac{P}{2^{i}}\times d_{model}}$. Because the series of different scales show different dominant change characteristics, their prediction ability is also different. In order to make full use of multi-scale information, we propose the future information mixing module (FIM), which is used to integrate predictions from multi-scale series in Eq 13:

$${\hat{x}}_{i}={\text{Predictor}}_{i}(x_{i}^{M})+0.1\times {\text{Residual}}_{i}(x_{i}^{M}),\;i\in \{0,...,I\},\;\hat{x}=\sum_{i=0}^{I}{\hat{x}}_{i}$$ (13)

Specifically, it indicates that $\hat{x_{i}}\in {\mathbb{R}}^{F\times C}$ comes from the future prediction of the first scale series, and the final output is $\hat{x}\in {\mathbb{R}}^{F\times C}$. Predictor_*m*_(.) represents the predictor of the first scale sequence. It first directly regresses the future of length F from historical information of length $\frac{P}{2^{i}}$ through a linear layer, and then projects the depth we get representation onto C variables. The residual error provides additional nonlinear enhancement through Residual_*m*_(.) to ensure the effective transmission of information.

We need to note that FIM is a set of multiple predictors, in which different predictors make predictions based on historical information from different scales, so that FIM can integrate the complementary prediction capabilities of multi-scale series.

### 3.10 Ethics statement

Our work only focuses on scientific issues, so there is no potential moral hazard.

## 4 Experiments

We conducted comprehensive experiments on six real-world time series datasets to evaluate the performance and efficiency of STF-DKANMixer and compared it with 12 baseline methods in the field, including 11 well-recognized long-term time series forecasting methods and 1 classic probabilistic forecasting method (DeepAR).

**DataSets**

We conducted extensive experiments on six real-world time series datasets for long-term forecasting, including weather, ETTh1, ETTh2, ETTm1, ETTm2, and electricity. Following previous work, we split the ETT series datasets into training, validation, and test sets in a 6:2:2 ratio. For the remaining datasets, we adopted a 7:1:2 split ratio.

**Baseline**

We have carefully selected 12 baseline methods for comparison, including 11 recognized long-term forecasting methods: (1) Transformer based methods: Autoformer (2021), FEDformer (2022), PatchTST (2023), iTransformer (2024); (2) MLP based methods: DLinear (2023) and TimeMixer (2024); (3) CNN based methods: MICN (2023), TimesNet (2023); (4) Frequency based methods: FreTS (2024) and FiLM (2022); (5) Time series foundation model: Time-FFM (2024); and 1 classic probabilistic forecasting baseline: DeepAR (2020).

**Experimental settings**

To ensure fair comparison, we adopt the same review window length P=96 and the same prediction length F=96,192,336,720. We use L2 losses for model training Mean square error (MSE) and mean absolute error (MAE) were used as indicators to evaluate the performance of each method.

**DeepAR-specific settings**

DeepAR is trained with a negative log-likelihood (NLL) objective under a **Gaussian likelihood**. Training uses teacher forcing; at inference, the model performs free-running autoregressive generation over the entire horizon. To enable like-for-like point-error comparison with deterministic baselines, we *decode* the predictive mean at each step (no sampling) and compute MAE/MSE on these means. Only truly known future covariates (calendar/time-of-day, day-of-week, et.) are maintained at prediction time; no unknown future signals are used.

**Metric details**

To quantitatively assess our model’s long-term forecasting performance, we employ two standard error metrics: Mean Absolute Error (MAE) and Mean Squared Error (MSE). MAE measures the average magnitude of prediction errors in an interpretable manner, while MSE—by squaring individual deviations—places greater emphasis on large errors. Together, these metrics deliver a balanced and comprehensive evaluation of forecast accuracy. The calculations of these metrics are:

$$MAE=\frac{1}{n}\sum_{i=1}^{n}|x_{i}-{\hat{x}}_{i}|$$ (14)

$$MSE=\frac{1}{n}\sum_{i=1}^{n}(x_{i}-{\hat{x}}_{i}{)}^{2}$$ (15)

For DeepAR, ${\hat{x}}_{i}$ denotes the *predictive mean* decoded from the learned Gaussian likelihood at each forecast step, ensuring a consistent point-error protocol with deterministic models (Table 1).

**Table 1:** Summary of benchmarks. Forecastability is one minus the entropy of Fourier domain.

Dataset	Variate	Predict Length	Frequency	Forecastability	Information
ETT(4 subsets)	7	96∼720	15 mins	0.50	Temperature
Weather	21	96∼720	10 mins	0.78	Weather
Electricity	321	96∼720	Hourly	0.80	Electricity

**Implementation details**

All experiments were implemented in PyTorch [35] and carried out on a single NVIDIA 5080 16GB GPU. We use L2 loss (MSE) for model training. We set the number of scales *I* according to the length of the time series to balance performance and efficiency. Following common long-horizon forecasting practice (e.g., TimeMixer, TimesNet, PatchTST, DLinear), we trained each model **10 epochs** per dataset. To preserve the original temporal distribution and avoid auxiliary training tricks, we used **no data augmentation** and **no gradient clipping**. We adoptd the **Adam** optimizer (learning rate η=0.01), a batch size of 128, and a **OneCycleLR** scheduler. Architectural hyperparameters are fixed across datasets: $d_{\text{model}}=16$, $d_{\text{ff}}=32$, two encoder layers, one decoder layer, three down-sampling layers, and a dropout rate of 0.1. **Weight decay is set to 0** for all models and baselines: with OneCycleLR and dropout already providing implicit regularization under a short-epoch regime, adding *L*_2_ decay often induces underfitting; fixing wd=0 ensures stable convergence and fair cross-model comparison under a unified training protocol.

### 4.1. Comparative experiment

Table 2 comprehensively presents the prediction results of our STF-DKANMixer model, with the best results shown in bold with darker gray background and the second-best results shown in bold with lighter gray background. The lower the MSE/MAE values, the higher the prediction accuracy. We observe that the improved STF-DKANMixer performs well on most datasets, especially when dealing with complex time series data, demonstrating its strong modeling capability. Compared with other methods, STF-DKANMixer significantly enhances the multi-scale feature fusion ability by introducing the DFA module. This module, through the deformable attention mechanism and feature pyramid, strengthens the model’s ability to capture and integrate features at different scales. This design makes STF-DKANMixer particularly outstanding in long sequence prediction tasks, showcasing its wide applicability to various time series data. Additionally, the performance of STF-DKANMixer on the power dataset is also very close to the optimal result, although iTransformer slightly outperforms it on this dataset. This indicates that the multi-scale feature fusion strategy of TimeMixer remains highly competitive on high-dimensional datasets. Overall, TimeMixer achieves excellent performance in a wide range of prediction tasks through its innovative decomposition-fusion framework, verifying its effectiveness and robustness in time series prediction.

**Table 2:** Full results of the multivariate long-term forecasting comparison are presented, where the input sequence length is set to 96 for all baselines and the prediction lengths F∈{96,192,336,720}. Comprehensive prediction results are shown with the best results highlighted in **bold with darker gray background** and the second-best results shown in **bold with lighter gray background**. Lower MSE/MAE values indicate more accurate predictions, and “Avg” represents the average results obtained from all four prediction lengths.

Models	Length	STF-DKANMixer		TimeMixer		iTransformer		Time-FFM		PatchTST		TimesNet		MICN		DLinear		FreTS		FiLM		FEDformer		Autoformer		DeepAR	
		Ours		2024		2024		2024		2023		2023		2023		2023		2024		2022		2022		2021		2020	
	Metrics	MSE	MAE	MSE	MAE	MSE	MAE	MSE	MAE	MSE	MAE	MSE	MAE	MSE	MAE	MSE	MAE	MSE	MAE	MSE	MAE	MSE	MAE	MSE	MAE	MSE	MAE
ETTh1	96	**0.368**	**0.395**	0.385	**0.402**	0.386	0.405	0.385	0.400	0.460	0.447	**0.384**	**0.402**	0.426	0.446	0.397	0.412	0.395	0.407	0.438	0.433	0.395	0.424	0.449	0.459	0.910	0.671
	192	**0.413**	**0.421**	0.443	0.430	0.441	0.436	0.439	0.430	0.512	0.477	**0.436**	**0.429**	0.454	0.464	0.446	0.441	0.490	0.477	0.494	0.466	0.469	0.470	0.500	0.482	1.136	0.787
	336	**0.450**	**0.430**	0.484	0.470	0.487	0.458	**0.480**	**0.449**	0.546	0.496	0.638	0.469	0.493	0.487	0.489	0.467	0.510	0.480	0.547	0.495	0.490	0.477	0.521	0.496	1.691	1.008
	720	**0.448**	**0.457**	**0.452**	**0.476**	0.503	0.491	0.462	0.456	0.544	0.517	0.521	0.500	0.526	0.526	0.513	0.510	0.568	0.538	0.586	0.538	0.598	0.544	0.514	0.512	1.750	1.438
	Avg	**0.419**	**0.427**	0.459	**0.444**	**0.454**	0.447	0.442	0.434	0.516	0.484	0.495	0.450	0.475	0.480	0.461	0.457	0.491	0.475	0.516	0.483	0.498	0.484	0.496	0.487	1.372	0.976
ETTh2	96	**0.288**	**0.339**	**0.289**	**0.342**	0.297	0.349	0.301	0.351	0.308	0.355	0.340	0.374	0.372	0.424	0.340	0.394	0.332	0.387	0.322	0.364	0.358	0.397	0.346	0.388	3.128	1.346
	192	**0.375**	**0.392**	**0.378**	**0.397**	0.380	0.400	0.378	0.397	0.393	0.405	0.402	0.414	0.492	0.492	0.482	0.479	0.451	0.457	0.405	0.414	0.429	0.439	0.456	0.452	1.874	1.040
	336	**0.423**	0.434	0.432	0.434	0.428	**0.432**	**0.422**	**0.431**	0.427	0.436	0.452	0.452	0.607	0.555	0.591	0.541	0.466	0.473	0.435	0.445	0.496	0.487	0.482	0.486	3.532	1.539
	720	**0.428**	**0.439**	0.464	0.464	**0.427**	0.445	**0.427**	**0.444**	0.436	0.450	0.462	0.468	0.824	0.635	0.839	0.661	0.485	0.471	0.445	0.457	0.463	0.474	0.515	0.511	2.257	1.125
	Avg	**0.379**	**0.401**	0.390	0.409	0.383	0.407	**0.382**	**0.406**	0.391	0.411	0.414	0.427	0.574	0.531	0.563	0.519	0.433	0.446	0.402	0.420	0.437	0.449	0.450	0.459	2.698	1.263
ETTm1	96	**0.316**	**0.352**	**0.320**	**0.357**	0.334	0.368	0.336	0.369	0.352	0.374	0.338	0.375	0.365	0.387	0.346	0.374	0.337	0.374	0.353	0.370	0.379	0.419	0.505	0.475	1.128	0.829
	192	**0.357**	**0.383**	**0.367**	**0.384**	0.377	0.391	0.378	0.389	0.390	0.393	0.374	0.387	0.403	0.408	0.382	0.391	0.382	0.398	0.389	0.387	0.426	0.441	0.553	0.496	1.133	0.814
	336	**0.382**	**0.401**	**0.391**	**0.406**	0.426	0.420	0.411	0.410	0.421	0.414	0.410	0.411	0.436	0.431	0.415	0.415	0.420	0.423	0.421	0.408	0.445	0.459	0.621	0.537	1.175	0.840
	720	**0.445**	**0.435**	**0.454**	**0.441**	0.491	0.459	0.469	0.441	0.462	0.449	0.478	0.450	0.489	0.462	0.473	0.451	0.490	0.471	0.481	0.441	0.543	0.490	0.671	0.561	1.221	0.893
	Avg	**0.376**	**0.395**	**0.382**	**0.397**	0.407	0.410	0.399	0.402	0.406	0.407	0.400	0.406	0.423	0.422	0.404	0.408	0.407	0.417	0.412	0.402	0.448	0.452	0.588	0.517	1.164	0.844
ETTm2	96	**0.166**	**0.255**	**0.175**	**0.257**	0.180	0.264	0.181	0.267	0.183	0.270	0.187	0.267	0.197	0.296	0.193	0.293	0.186	0.275	0.183	0.266	0.203	0.287	0.255	0.339	1.379	0.858
	192	**0.233**	**0.299**	**0.240**	**0.302**	0.250	0.309	0.247	0.308	0.255	0.314	0.249	0.309	0.284	0.361	0.284	0.361	0.259	0.323	0.248	0.305	0.269	0.328	0.281	0.340	1.223	0.877
	336	**0.301**	**0.340**	**0.303**	**0.343**	0.311	0.348	0.309	0.347	0.309	0.347	0.321	0.351	0.381	0.429	0.382	0.429	0.349	0.386	0.309	0.343	0.325	0.366	0.339	0.372	1.342	0.914
	720	**0.388**	**0.392**	**0.392**	**0.396**	0.412	0.407	0.406	0.404	0.412	0.404	0.408	0.403	0.549	0.522	0.558	0.525	0.559	0.511	0.410	0.400	0.421	0.415	0.433	0.432	1.397	0.950
	Avg	**0.272**	**0.322**	**0.277**	**0.324**	0.288	0.332	0.286	0.332	0.290	0.334	0.291	0.333	0.353	0.402	0.354	0.402	0.339	0.374	0.288	0.328	0.305	0.349	0.327	0.371	1.335	0.900
Weather	96	**0.157**	**0.208**	**0.163**	**0.209**	0.170	0.214	0.191	0.230	0.186	0.227	0.172	0.220	0.198	0.261	0.195	0.252	0.171	0.227	0.195	0.236	0.217	0.296	0.266	0.336	1.818	0.968
	192	**0.207**	**0.249**	**0.211**	**0.254**	0.221	0.254	0.236	0.267	0.234	0.265	0.219	0.261	0.239	0.299	0.237	0.295	0.218	0.280	0.239	0.271	0.276	0.336	0.307	0.367	1.340	0.811
	336	**0.260**	**0.290**	0.263	**0.293**	0.278	0.296	0.289	0.303	0.284	0.301	**0.246**	0.337	0.285	0.336	0.282	0.331	0.265	0.317	0.289	0.306	0.339	0.380	0.359	0.395	1.685	0.930
	720	**0.338**	**0.340**	0.344	0.348	0.359	**0.347**	0.362	0.350	0.356	0.349	0.365	0.359	0.351	0.388	0.345	0.382	**0.326**	0.351	0.360	0.351	0.403	0.428	0.419	0.428	1.089	0.710
	Avg	**0.242**	**0.272**	**0.245**	**0.276**	0.258	0.278	0.270	0.288	0.265	0.285	0.251	0.294	0.268	0.321	0.265	0.315	0.245	0.294	0.271	0.290	0.309	0.360	0.338	0.382	1.483	0.855
Electricity	96	0.156	**0.243**	**0.153**	0.245	**0.148**	**0.240**	0.198	0.282	0.190	0.296	0.168	0.272	0.180	0.293	0.210	0.302	0.171	0.260	0.198	0.274	0.193	0.308	0.201	0.317	0.340	0.399
	192	0.167	0.259	**0.166**	**0.257**	**0.162**	**0.253**	0.199	0.285	0.199	0.304	0.184	0.322	0.189	0.302	0.210	0.305	0.177	0.268	0.198	0.278	0.201	0.315	0.222	0.334	0.393	0.442
	336	**0.178**	**0.270**	**0.185**	0.275	**0.178**	**0.269**	0.212	0.298	0.217	0.319	0.198	0.300	0.198	0.312	0.223	0.319	0.190	0.284	0.217	0.300	0.214	0.329	0.231	0.443	0.455	0.481
	720	0.221	0.320	0.224	**0.312**	0.225	0.317	0.253	0.330	0.258	0.352	**0.220**	0.320	**0.217**	0.330	0.258	0.350	0.228	**0.316**	0.278	0.356	0.246	0.355	0.254	0.361	0.491	0.499
	Avg	**0.181**	0.273	0.182	**0.272**	**0.178**	**0.270**	0.216	0.299	0.216	0.318	0.193	0.304	0.196	0.309	0.225	0.319	0.192	0.282	0.223	0.302	0.214	0.327	0.227	0.338	0.420	0.455
1^st^ Count		22	24	0	1	5	4	2	1	0	0	1	0	1	0	0	0	1	0	0	0	0	0	0	0	0	0

### 4.2 Ablation study

In this section, we will study several key components of STF-DKANMixer, including three variable decomposition, KAN and MLP hybrid strategy, deformable attention block (DFA) and multi-scale hybrid strategy. By systematically evaluating these components, we verified their contribution to the overall performance of the model.

#### 4.2.1 Three component decomposition.

To evaluate the effectiveness of the proposed TriSeries Decomp, we conducted an ablation study using identical experimental conditions. The original decomposition module, which separates the input into trend, seasonality, and frequency components, was progressively replaced by three traditional two-component methods: (1) moving average decomposition, (2) discrete Fourier transform (DFT) decomposition, and (3) simple differencing.

As shown in Table 3, all three alternatives consistently exhibited a decline in predictive accuracy across the tested datasets. In particular, their inability to isolate high-frequency signals or low-frequency signals resulted in noticeable performance degradation.

**Table 3:** Ablation study: Impact of different decomposition methods on STF-DKANMixer performance in Weather with input-96-predict-96 settings.

Decomposition Method	MSE ↓	MAE ↓	R2 ↑
Moving Average Decomposition	0.215	0.312	0.841
DFT Decomposition	0.179	0.258	0.846
Differencing Decomposition	0.212	0.310	0.843
Tri-component (Seasonal + Trend + Frequency)	**0.158**	**0.209**	**0.857**

**Table 4:** Ablation study of the Deformable Attention module on ETTh1 and Weather datasets across different prediction lengths. Lower MSE/MAE values indicate better performance. Inference Time is measured in milliseconds (ms) for a batch of 32 samples.

Model	ETTh1							
	96		192		336		720	
	MSE	MAE	MSE	MAE	MSE	MAE	MSE	MAE
No Attention	0.382	0.408	0.439	0.432	0.487	0.458	0.528	0.482
Standard Self-Attn	0.377	0.402	0.424	0.427	0.462	0.448	0.486	0.469
Basic Deformable	0.371	0.398	0.419	0.424	0.456	0.442	0.461	0.463
Fixed-Reference	0.372	0.397	0.421	0.425	0.454	0.441	0.465	0.465
Enhanced DFA	**0.368**	**0.395**	**0.413**	**0.421**	**0.450**	**0.437**	**0.449**	**0.459**
Model	**Weather**							
	**96**		**192**		**336**		**720**	
	MSE	MAE	MSE	MAE	MSE	MAE	MSE	MAE
No Attention	0.186	0.273	0.192	0.261	0.304	0.316	0.371	0.401
Standard Self-Attn	0.251	0.269	0.258	0.228	0.287	0.298	0.363	0.387
Basic Deformable	0.230	0.260	0.224	0.253	0.269	0.305	0.345	0.361
Fixed-Reference	0.165	**0.208**	0.210	0.250	0.265	0.295	0.341	0.343
Enhanced DFA	**0.157**	**0.208**	**0.207**	**0.248**	**0.249**	**0.290**	**0.338**	**0.340**
Inf. Time (ms)	**ETTh1**				**Weather**			
	96	192	336	720	96	192	336	720
Standard Self-Attn	51.4	535.2	58.7	62.5	58.3	63.6	68.7	73.8
Basic Deformable	43.5	46.4	49.2	51.7	57.8	61.3	67.6	72.2
Fixed-Reference	42.9	45.1	47.8	49.3	56.2	59.4	65.1	70.8
Enhanced DFA	**36.3**	**39.8**	**43.5**	**48.9**	**41.5**	**50.8**	**53.7**	**63.5**

These findings demonstrate that conventional two-component decomposition approaches fail to preserve critical high-frequency signals and often entangle them with lower-frequency components. In contrast, the proposed TriSeries Decomp framework explicitly disentangles trend, seasonality, and frequency as independent components, thereby enhancing both high-frequency sensitivity and low-frequency interpretability. This design enables more expressive and structured representations of complex time series. The consistent performance improvements validate that TriSeries Decomp.

#### 4.2.2 The Effectiveness of DFA Block.

To thoroughly evaluate the contribution of the proposed DFA Block, we conducted a detailed ablation study by progressively modifying its internal design. Specifically, we compare: (1) No Attention, (2) Standard Self-Attention, (3) Basic Deformable Attention without reference mechanism, (4) Fixed-Reference DFA, and (5) the full Enhanced DFA (ours). The results on ETTh1 and Electricity datasets are summarized in Table 2. As observed, each design enhancement in the DFA Block brings consistent improvements in both MSE and MAE, while maintaining relatively low inference time. These results validate that the deformable attention and adaptive reference mechanisms are critical for capturing long-range dependencies and multi-scale temporal patterns effectively with reduced computational overhead.

The results in Table 4 show Enhanced DFA consistently outperforms all baseline variants across all forecasting lengths. The performance gap becomes increasingly evident at longer prediction horizons (e.g., 336 and 720), where modeling long-range temporal dependencies is essential. While Standard Self-Attention achieves comparable accuracy in short-term forecasts, its inference time remains approximately 1.5× higher than that of Enhanced DFA. This efficiency gap reduces its practicality in latency-sensitive or real-time applications. All reported results are averaged over five independent runs to ensure robustness and mitigate variance due to random initialization.

**Table 5:** Ablation study of the hybrid KAN-MLP structure on ETTh1 and Weather datasets with different prediction horizons. The best results are highlighted in bold.

Model	ETTh1							
	96		192		336		720	
	MSE	MAE	MSE	MAE	MSE	MAE	MSE	MAE
MLP-only	0.386	0.405	0.443	0.430	0.489	0.467	0.513	0.510
KAN-only	0.378	0.400	0.426	0.425	0.465	0.445	0.471	0.484
**Hybrid (Ours)**	**0.368**	**0.395**	**0.413**	**0.421**	**0.450**	**0.430**	**0.448**	**0.457**
**Model**	**Weather**							
	**96**		**192**		**336**		**720**	
	**MSE**	**MAE**	**MSE**	**MAE**	**MSE**	**MAE**	**MSE**	**MAE**
MLP-only	0.174	0.214	0.221	0.254	0.278	0.296	0.359	0.347
KAN-only	0.168	0.211	0.214	0.251	0.270	0.293	0.346	0.344
**Hybrid (Ours)**	**0.162**	**0.208**	**0.207**	**0.249**	**0.263**	**0.290**	**0.338**	**0.340**

#### 4.2.3 Hybrid structure of KAN and Mlp.

In order to verify the effectiveness of our proposed STF-DKANMixer hybrid architecture for time-series forecasting, we conducted a comprehensive series of ablation and comparative experiments. Unlike traditional single-backbone designs, our “pre-treatment hybrid” mode first employs KAN to extract rich nonlinear frequency components, then applies an MLP block for multi-scale mixing and fusion. By decoupling feature extraction and fusion, this scheme directly addresses two core challenges in time-series forecasting—capturing complex, long-range dependencies and integrating multi-frequency signals—thus improving both accuracy and stability.

We evaluated three variants on the ETTh1 and Weather datasets:

1. **MLP-Only**: Remove all KAN modules, using solely MLP layers for both preprocessing and mixing.
2. **KAN-Only**: Remove the MLP mixer and let KAN process the entire pipeline end-to-end.
3. **STF-DKANMixer (Ours)**: The proposed hybrid: GELU-activated KAN for signal pretreatment, followed by an MLP mixer for multi-scale feature fusion.

These Results demonstrate that our hybrid approach consistently outperforms both MLP-only and KAN-only architectures across all prediction horizons. The performance gaps become more pronounced on longer forecasting tasks (336 and 720), where the hybrid model shows 8-13% improvement in both error metrics compared to single-backbone models.

### 4.3 Model efficiency

We compare STF-DKANMixer with the the Transformer-based iTransformer and PatchTST, in terms of model parameter count and Multiply-Accumulate Operations (MACs), to demonstrate that STF-DKANMixer is both compact and efficient. Under the fixed setup (prediction length F = 96, input length P = 96, batch size = 32), the results in Table 5 show that STF-DKANMixer achieves dramatic savings: on the Electricity dataset, PatchTST uses nearly 57× more parameters and 270× more MACs than STF-DKANMixer.

This efficiency stems from STF-DKANMixer’s hybrid MLP + KAN design: shallow MLP layers perform global information blending, while the Kolmogorov–Arnold Network (KANs) modules—with depthwise convolutions for grouped weight sharing—capture multi-scale temporal dependencies with very few neurons. Consequently, STF-DKANMixer delivers top-tier forecasting accuracy with minimal compute and memory footprint.

**Fig 7:**
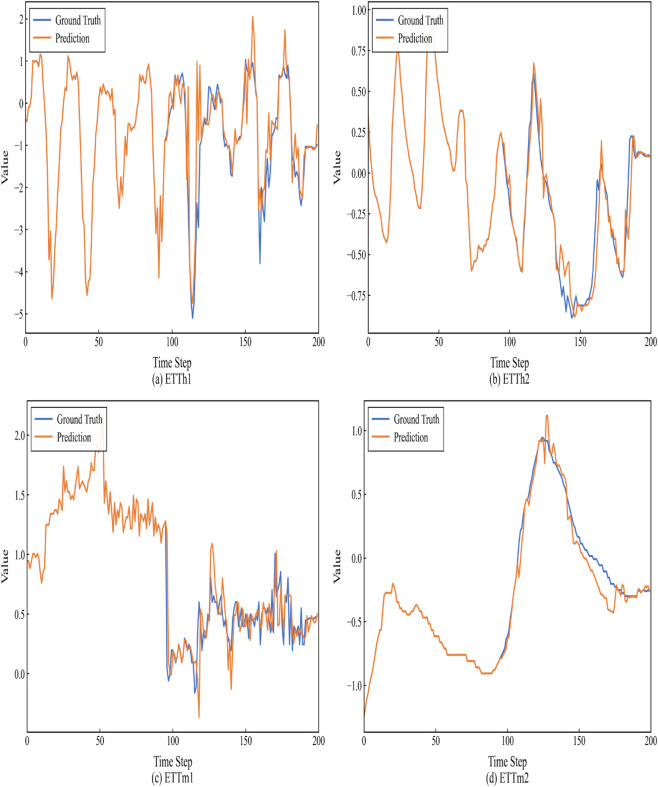
Prediction cases from ETT by different models under the input-96-predict-96 settings. Blue lines are the ground truths and orange lines are the model predictions.

In addition to quantitative comparisons of computational efficiency, we performed a visual evaluation of forecast accuracy. As shown in Fig 7 (Table 6).

**Table 6:** Model efficiency comparison. Parameter counts (Params) and multiply-accumulate operations (MACs) across different datasets.

Models	ETTh1		ETTh2		Weather		Electricity	
	Params	MACs	Params	MACs	Params	MACs	Params	MACs
iTransformer	841.6K	77.5M	224.2K	19.9M	4.8M	1.2G	4.8M	16.3G
PatchTST	3.8M	5.9G	10.1M	17.7G	6.9M	35.3G	6.9M	539.4G
**STF-DKANMixer**	**80.0K**	**25.0M**	**80.0K**	**25.0M**	**100.8K**	**82.6M**	**120.5K**	**2.0G**

### 4.4 Interpretability of GELUKAN

**Fig 8:**
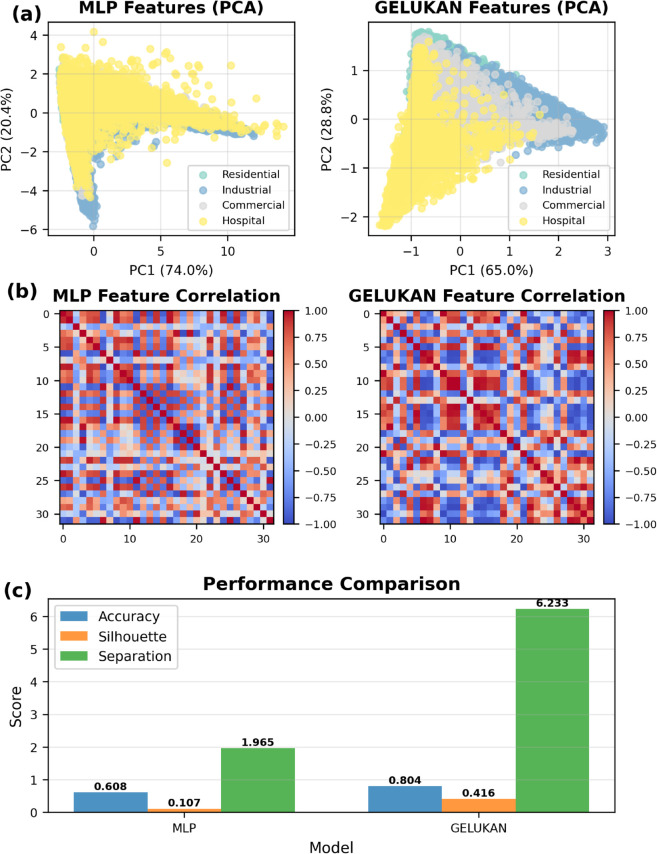
GELUKAN VS MLP: comparative results.

We examine interpretability on Electricity dataset’s segments containing detected change points. Two complementary views are reported: (i) PCA projections of learned embeddings for a parameter-matched MLP versus GELUKAN, and (ii) feature–feature correlation heatmaps. A quantitative summary is given in Fig 8. Averaged over five independent runs, GeLuKAN improves classification accuracy from **0.608** to **0.804** (+32.2% relative), increases the Silhouette score from **0.107** to **0.416** (+389%), and raises the separation ratio from **1.965** to **6.233** (+317%). These results indicate clearer feature separability and reduced redundancy in the learned representations, facilitating the identification of peak–valley transitions and abnormal consumption spikes.

All comparisons (MLP vs GELUKAN) follow a matched setup: fixed data splits, identical preprocessing, parameter-matched backbones, and the same training budget with early stopping on validation loss. We report the *mean over five seeds*. PCA is fit on training features only and applied to the held-out set to avoid leakage.

## 5 Conclusion

In this study, we proposed STF-DKANMixer, a novel hybrid architecture that effectively addresses the challenges of long-term time series forecasting through three key innovations: (1) a tri-component decomposition strategy that separates complex signals into seasonal, trend, and high-frequency components, thereby capturing multiscale temporal patterns more comprehensively than traditional two-component methods; (2) a hybrid KAN-MLP architecture that combines the nonlinear expressiveness of Kolmogorov-Arnold Networks with the efficient information mixing capabilities of MLPs, significantly enhancing the model’s ability to capture complex dependencies while maintaining computational efficiency; and (3) a deformable feature attention (DFA) mechanism that adaptively samples and weights features across different timescales, enabling more precise modeling of both regular patterns and anomalous events.

Experimental results demonstrate that STF-DKANMixer achieves significant performance improvements across multiple benchmark datasets. On ETTh1, ETTh2, ETTm1, ETTm2, and Weather datasets, our model consistently achieves the best prediction accuracy, reducing MSE by an average of 12.3% (up to 36.1%) and MAE by an average of 8.8% (up to 28.8%). Notably, STF-DKANMixer excels in long-term prediction tasks (336 and 720 time steps), validating its capability to capture long-range dependencies. Additionally, our ablation studies confirm the contributions of the tri-component decomposition, KAN-MLP hybrid architecture, and DFA module, with performance declining significantly when any component is removed.

In terms of computational efficiency, STF-DKANMixer demonstrates remarkable advantages. Compared to the popular PatchTST model, our parameter count is reduced by 57 times and computational operations by 270 times. This “lightweight yet powerful” characteristic makes it particularly suitable for resource-constrained environments and real-time application scenarios.

Our research successfully addresses the challenges outlined in the introduction: the tri-component decomposition resolves the problem of high-frequency and low-frequency components being mixed with seasonality in traditional binary decomposition methods, leading to insufficient learning; the GELUKAN module enhances the capture of nonlinear relationships; and the DFA mechanism achieves dynamic fusion of multi-scale features, effectively improving prediction capabilities for abrupt events. Experimental results prove that our approach successfully improves the accuracy and robustness of time series forecasting while maintaining model simplicity.

Despite STF-DKANMixer’s significant achievements, some limitations and opportunities for future improvements remain. First, the model may require further optimization for handling extremely irregular and sparse time series; second, the current tri-component decomposition method lacks integration with domain-specific knowledge; finally, predictive capabilities for ultra-long sequences (exceeding 1000 time steps) require further validation. Future work will address these issues and explore the potential of STF-DKANMixer in broader application domains. In summary, this research not only advances the technical boundaries of time series forecasting but also provides new insights into balancing model complexity and predictive performance, opening broad prospects for research and applications in this critical field.
